# Solving the multiple-set split equality common fixed-point problem of firmly quasi-nonexpansive operators

**DOI:** 10.1186/s13660-018-1668-0

**Published:** 2018-04-13

**Authors:** Jing Zhao, Haili Zong

**Affiliations:** 1 Tianjin Key Lab for Advanced Signal Processing, Civil Aviation University of China, Tianjin, China; 2 College of Science, Civil Aviation University of China, Tianjin, China

**Keywords:** 47H09, 47H10, 47J05, 54H25, The multiple-set split equality common fixed-point problem, The multiple-set split equality problem, Firmly quasi-nonexpansive mapping, Weak convergence, Iterative algorithms, Hilbert space

## Abstract

In this paper, we propose parallel and cyclic iterative algorithms for solving the multiple-set split equality common fixed-point problem of firmly quasi-nonexpansive operators. We also combine the process of cyclic and parallel iterative methods and propose two mixed iterative algorithms. Our several algorithms do not need any prior information about the operator norms. Under mild assumptions, we prove weak convergence of the proposed iterative sequences in Hilbert spaces. As applications, we obtain several iterative algorithms to solve the multiple-set split equality problem.

## Introduction

Let $H_{1}$, $H_{2}$, and $H_{3}$ be real Hilbert spaces. The multiple-set split equality common fixed-point problem (MSECFP) is to find $x^{*}, y^{*}$ with the property

1.1 $$ x^{*} \in \bigcap^{p}_{i=1}F(U_{i}), \qquad y^{*}\in \bigcap_{j=1}^{r}F(T_{j}) \quad \text{such that } Ax^{*} = By^{*}, $$

where $p,r\geq1$ are integers, $\{U_{i}\}_{i=1}^{p}:H_{1}\rightarrow H_{1} $ and $\{T_{j}\}^{r}_{j=1}:H_{2}\rightarrow H_{2} $ are nonlinear operators, $A: H_{1}\rightarrow H_{2}$ and $B: H_{2}\rightarrow H_{3}$ are two bounded linear operators. If $U_{i}$ $(1\leq i\leq p)$ and $T_{j}$ $(1\leq j\leq r)$ are projection operators, then the MSECFP is reduced to the multiple-set split equality problem (MSEP):

1.2 $$ \mbox{finding}\quad x^{*} \in \bigcap^{p}_{i=1}C_{i} \quad \text{and}\quad y^{*}\in \bigcap_{j=1}^{r}Q_{j} \quad \text{such that } Ax^{*} = By^{*}, $$

where $\{C_{i}\}_{i=1}^{p}$ and $\{Q_{j}\}_{j=1}^{r}$ are nonempty closed convex subsets of real Hilbert spaces $H_{1}$ and $H_{2}$, respectively. When $p=r=1$, the MSECFP and MSEP become the split equality common fixed-point problem (SECFP) and split equality problem (SEP), respectively, which were first put forward by Moudafi [1]. These allow asymmetric and partial relations between the variables *x* and *y*. They are applied in many situations, for instance, in game theory and in intensity-modulated radiation therapy (see [2] and [3]).

If $H_{2}=H_{3}$ and $B=I$, then MSECFP (1.1) reduces to the multiple-set split common fixed-point problem (MSCFP):

1.3 $$ \mbox{finding}\quad x^{*}\in \bigcap^{p}_{i=1} F(U_{i})\quad \text{such that } Ax^{*}\in \bigcap ^{r}_{j=1} F(T_{j}) $$

and MSEP (1.2) reduces to the multiple-set split feasibility problem (MSFP):

1.4 $$ \mbox{finding}\quad x^{*} \in \bigcap^{p}_{i=1}C_{i} \quad \text{such that } Ax^{*}\in \bigcap_{j=1}^{r}Q_{j}. $$

They play significant roles in dealing with problems in image restoration, signal processing, and intensity-modulated radiation therapy [3–6]. With $p=r=1$, MSCFP (1.3) is known as the split common fixed-point problem (SCFP) and MSFP (1.4) is known as the split feasibility problem (SFP). Many iterative algorithms have been developed to solve the MSCFP and the MSFP. See, for example, [7–14] and the references therein.

Note that the SFP can be formulated as a fixed-point equation

1.5 $$ P_{C} \bigl(I-\gamma A^{\ast}(I-P_{Q})A \bigr)x^{\ast}= x^{\ast}, $$

where $P_{C}$ and $P_{Q}$ are the (orthogonal) projections onto *C* and *Q*, respectively, $\gamma>0$ is any positive constant, and $A^{\ast}$ denotes the adjoint of *A*. This implies that we can use fixed-point algorithms (see [15–21]) to solve SFP. Byrne [22] proposed the so-called CQ algorithm which generates a sequence $\{x_{k}\}$:

1.6 $$ x_{n+1}=P_{C} \bigl(x_{n}-\gamma A^{\ast}(I-P_{Q})Ax_{n} \bigr), $$

where $\gamma \in (0,2/ \lambda)$ with *λ* being the spectral radius of the operator $A^{\ast}A$. The CQ algorithm is efficient when $P_{C}$ and $P_{Q}$ are easily calculated. However, if *C* and *Q* are complex sets, for example, the fixed-point sets, the efficiency of the CQ algorithm will be affected because the projections onto such convex sets are generally hard to be accurately calculated. To solve the SCFP of nonexpansive operators, Censor and Segal [23] proposed and proved, in finite-dimensional spaces, the convergence of the following algorithm:

1.7 $$ x_{n+1}=U \bigl(x_{n}-\gamma A^{\ast}(I-T)Ax_{n} \bigr),\quad n\in N, $$

where $\gamma\in(0, \frac{2}{\lambda})$ with *λ* being the largest eigenvalue of the matrix $A^{\ast}A$.

For solving the constrained MSFP, Censor et al. [6] introduced the following proximity function:

1.8 $$ g(x):=\frac{1}{2}\sum_{i=1}^{p} \alpha_{i} \Vert x-P_{C_{i}}x \Vert ^{2}+ \frac{1}{2}\sum_{j=1}^{r} \beta_{j} \bigl\Vert Ax-P_{Q_{j}}(Ax) \bigr\Vert ^{2}, $$

where $\alpha_{i}>0$ $(1\leq i\leq p)$, $\beta_{j}>0$ $(1\leq j\leq r)$, and $\sum_{i=1}^{p}\alpha_{i}+\sum_{j=1}^{r} \beta_{j}=1$. Then

$$\nabla g(x)=\sum_{i=1}^{p} \alpha_{i}(x-P_{C_{i}}x)+\sum_{j=1}^{r} \beta_{j} A^{\ast}\bigl(Ax-P_{Q_{j}}(Ax) \bigr), $$

and they proposed the following projection method:

1.9 $$ x_{n+1}=P_{\Omega}\bigl(x_{n}-\gamma \nabla g(x_{n}) \bigr), $$

where Ω is the constrained set, $0<\gamma_{L}\leq \gamma\leq \gamma_{U}<\frac{2}{L}$, and *L* is the Lipschitz constant of ∇*g*.

For solving MSCFP (1.3) of directed operators, Censor and Segal [23] introduced a parallel iterative algorithm as follows:

1.10 $$ x_{n+1}=x_{n}-\gamma \Biggl[\sum _{i=1}^{p}\alpha_{i} \bigl(x_{n}-U_{i}(x_{n}) \bigr)+\sum_{j=1}^{r}\beta_{j}A^{\ast}\bigl(Ax_{n}-T_{j}(Ax_{n}) \bigr) \Biggr], $$

where $\{\alpha_{i}\}_{i=1}^{p}$, $\{\beta_{j}\}_{j=1}^{r}$ are nonnegative constants, $0<\gamma<2/L$ with $L = \sum_{i=1}^{p}\alpha_{i}+\lambda\sum_{j=1}^{r}\beta_{j}$ and *λ* being the largest eigenvalue of $A^{\ast}A$. They obtained the convergence of iterative algorithm (1.6).

Wang and Xu [24] proposed the following cyclic iterative algorithm for MSCFP (1.3) of directed operators:

1.11 $$ x_{n+1}=U_{[n]_{1}}(x_{n}+\gamma A^{\ast}(T_{[n]_{2}}-I)Ax_{n}, $$

where $0<\gamma< 2/\rho(A^{\ast}A)$, $[n]_{1} := n (\operatorname {mod}p)$, and $[n]_{2} := n (\operatorname {mod}r)$. They proved the weak convergence of the sequence $\{x_{n}\}$ generated by (1.7).

For solving MSCFP (1.3), Tang and Liu [25] introduced inner parallel and outer cyclic iterative algorithm:

1.12 $$ x_{n+1}=U_{[n]_{1}} \Biggl(x_{n}+ \gamma_{n}\sum_{j=1}^{r} \eta_{j}A^{*}(T_{j}-I)Ax_{n} \Biggr) $$

and outer parallel and inner cyclic iterative algorithm:

1.13 $$ x_{n+1}=\sum_{i=1}^{p} \omega_{i}U_{i} \bigl(x_{n}+\gamma_{n}A^{*}(T_{[n]_{2}}-I)Ax_{n} \bigr) $$

for directed operators $\{U_{i}\}_{i=1}^{p}$ and $\{T_{j}\}_{j=1}^{r}$, where $[n]_{1}=n(\operatorname {mod}p)$, $[n]_{2}=n(\operatorname {mod}r)$, $0< a\leq \gamma_{n}\leq b< 2/\rho(A^{\ast}A)$, $\{\eta_{j}\}_{j=1}^{r}$, $\{\omega_{i}\}_{i=1}^{p}\subset(0,1)$ with $\sum_{j=1}^{r}\eta_{j}=1$ and $\sum_{i=1}^{p}\omega_{i}=1$. They obtained the weak convergence of the above two mixed iterative sequences to solve MSCFP (1.3) of directed operators.

The SEP proposed by Moudafi [1] is to

1.14 $$ \mbox{find}\quad x^{*} \in C,\qquad y^{*}\in Q\quad \text{such that } Ax^{*} = By^{*}, $$

which can be written as the following minimization problem:

1.15 $$ \min_{x\in C,y\in Q}\frac{1}{2} \Vert Ax-By \Vert ^{2}. $$

Assume that the solution set of the SEP is nonempty. By the optimality conditions, Moudafi [1] obtained the following fixed-point formulation: $(x^{\ast},y^{\ast})$ solves the SEP if and only if

1.16 $$ \textstyle\begin{cases} x^{\ast}=P_{C}(x^{\ast}-\gamma A^{\ast}(Ax^{\ast}-By^{\ast})),\\ y^{\ast}=P_{Q}(y^{\ast}+\beta B^{\ast}(Ax^{\ast}-By^{\ast})), \end{cases} $$

where *γ*, $\beta>0$. Therefore, for solving the SECP of firmly quasi-nonexpansive operators, Moudafi [1] introduced the following alternating algorithm:

1.17 $$ \textstyle\begin{cases} x_{n+1}=U(x_{n}-\gamma_{n}A^{\ast}(Ax_{n}-By_{n})),\\ y_{n+1}=T(y_{n}+\gamma_{n}B^{\ast}(Ax_{n+1}-By_{n})), \end{cases} $$

where a nondecreasing sequence $\gamma_{n}\in (\varepsilon,\min(\frac{1}{\lambda_{A}},\frac{1}{\lambda_{B}})-\varepsilon)$, $\lambda_{A}$, $\lambda_{B}$ stand for the spectral radius of $A^{\ast}A$ and $B^{\ast}B$, respectively. In [26], Moudafi and Al-Shemas introduced the following simultaneous iterative method:

1.18 $$ \textstyle\begin{cases} x_{n+1}=U(x_{n}-\gamma_{n}A^{\ast}(Ax_{n}-By_{n})),\\ y_{n+1}=T(y_{n}+\gamma_{n}B^{\ast}(Ax_{n}-By_{n})), \end{cases} $$

where $\gamma_{n}\in(\varepsilon,\frac{2}{\lambda_{A}+\lambda_{B}}-\varepsilon)$, $\lambda_{A}$, $\lambda_{B}$ stand for the spectral radius of $A^{\ast}A$ and $B^{\ast}B$, respectively. Recently, many iterative algorithms have been developed to solve the SEP, SECFP, and MSEP. See, for example, [27–34] and the references therein. Note that in algorithms (1.17) and (1.18), the determination of the step size $\{\gamma_{n}\}$ depends on the operator (matrix) norms $\Vert A \Vert $ and $\Vert B \Vert $ (or the largest eigenvalues of $A^{\ast}A$ and $B^{\ast}B$). To overcome this shortage, we introduce parallel and cyclic iterative algorithms with self-adaptive step size to solve MSECFP (1.1) governed by firmly quasi-nonexpansive operators. We also propose two mixed iterative algorithms which combine the process of cyclic and parallel iterative methods and do not need the norms of bounded linear operators. As applications, we obtain several iterative algorithms to solve MSEP (1.2).

## Preliminaries

### Concepts

Throughout this paper, we always assume that *H* is a real Hilbert space with the inner product $\langle\cdot,\cdot\rangle$ and the norm $\Vert \cdot \Vert $. Let *I* denote the identity operator on *H*. Denote the fixed-point set of an operator *T* by $F(T)$. We denote by → the strong convergence and by ⇀ the weak convergence. We use $\omega_{w}(x_{k})=\{x:\exists x_{k_{j}} \rightharpoonup x\}$ to stand for the weak *ω*-limit set of $\{x_{k}\}$ and use Γ to stand for the solution set of MSECFP (1.1).

#### Definition 2.1

An operator $T:H\rightarrow H$ is said to be

(i) nonexpansive if $\Vert Tx-Ty \Vert \leq \Vert x-y \Vert $ for all $x,y\in H$;
(ii) firmly nonexpansive if $\Vert Tx-Ty \Vert ^{2}\leq \Vert x-y \Vert ^{2}- \Vert (x-y)-(Tx-Ty) \Vert ^{2}$ for all $x,y\in H$;
(iii) firmly quasi-nonexpansive (i.e., directed operator) if $F(T)\neq\emptyset$ and
  $$\Vert Tx-q \Vert ^{2}\leq \Vert x-q \Vert ^{2}- \Vert x-Tx \Vert ^{2} $$
  or equivalently
  $$\langle x-q,x-Tx\rangle\geq \Vert x-Tx \Vert ^{2} $$
  for all $x\in H$ and $q\in F(T)$.

#### Definition 2.2

An operator $T:H\rightarrow H$ is called demiclosed at the origin if, for any sequence $\{x_{n}\}$ which weakly converges to *x*, and if the sequence $\{Tx_{n}\}$ strongly converges to 0, then $Tx=0$.

Recall that the metric (nearest point) projection from *H* onto a nonempty closed convex subset *C* of *H*, denoted by $P_{C}$, is defined as follows: for each $x\in H$,

$$P_{C}(x)=\operatorname{arg}\min_{y\in C} \bigl\{ \Vert x-y \Vert \bigr\} . $$

It is well known that $P_{C}x$ is characterized by the inequality

$$P_{C}x\in C,\qquad \langle x-P_{C}x,z-P_{C}x \rangle\leq 0,\quad z\in C. $$

#### Remark 2.1

It is easily seen that a firmly nonexpansive operator is nonexpansive. Firmly quasi-nonexpansive operators contain firmly nonexpansive operators with a nonempty fixed-point set. A projection operator is firmly nonexpansive.

### Mathematical model

Recall that the SCFP is to find $x^{\ast}$ with the property

2.1 $$ x^{\ast}\in F(U)\quad \text{such that } Ax^{\ast}\in F(T) $$

and the SFP is to find $x^{\ast}$ with the property:

2.2 $$ x^{\ast}\in C\quad \text{such that } Ax^{\ast}\in Q, $$

where $A:\ H_{1}\rightarrow H_{2}$ is a bounded linear operator, $U:H_{1}\rightarrow H_{1} $ and $T:H_{2}\rightarrow H_{2} $ are nonlinear operators, *C* and *Q* are closed convex sets of Hilbert spaces $H_{1}$ and $H_{2}$, respectively.

We can formulate SFP (2.2) as an optimization. First, we consider the following proximity function:

$$g(x) = \frac{1}{2} \Vert x-P_{C}x \Vert ^{2}+ \frac{1}{2} \Vert Ax-P_{Q}Ax \Vert ^{2}. $$

Then the proximity function $g(x)$ is convex and differentiable with gradient

$$\nabla g(x) =x-P_{C}x+A^{\ast}(I-P_{Q})Ax, $$

where $A^{\ast}$ denotes the adjoint of *A*. Assume that the solution set of the SFP is nonempty, then $x^{\ast}$ is a solution of the SFP if and only if $x^{\ast}=\operatorname{arg}\min_{x\in H_{1}} g(x)$, i.e.,

$$\nabla g \bigl(x^{\ast}\bigr)=0, $$

which is equivalent to

2.3 $$ \begin{aligned}[b] x^{\ast}&=x^{\ast}-\tau\nabla g \bigl(x^{\ast}\bigr) \\ &=x^{\ast}-\tau \bigl(x^{\ast}-P_{C}x^{\ast}+A^{\ast}(I-P_{Q})Ax^{\ast}\bigr) \end{aligned} $$

for all $\tau>0$. For solving the SCFP of directed operators (i.e., firmly quasi-nonexpansive operators), Wang [35] proposed the following algorithm:

2.4 $$ x_{n+1}= x_{n}-\tau_{n} \bigl[(x_{n}-Ux_{n}) + A^{\ast}(I-T)Ax_{n} \bigr], $$

where the variable size step $\tau_{n}$ was chosen:

$$\tau_{n}=\frac{ \Vert x_{n}-Ux_{n} \Vert ^{2}+ \Vert (I-T)Ax_{n} \Vert ^{2}}{ \Vert (x_{n}-Ux_{n})+A^{\ast}(I-T)Ax_{n} \Vert ^{2}}. $$

This algorithm can be obtained by the fixed-point Eq. (2.3), where projection operators $P_{C}$ and $P_{Q}$ are replaced by *U* and *T*.

Setting

2.5 $$ h(x,y)=\frac{1}{2}\sum_{i=1}^{p} \alpha_{i} \Vert x-P_{C_{i}}x \Vert ^{2}+ \frac{1}{2}\sum_{j=1}^{r} \beta_{j} \Vert y-P_{Q_{j}}y \Vert ^{2}+ \frac{1}{2} \Vert Ax-By \Vert ^{2}, $$

MSEP (1.2) can be written as the following minimization problem:

$$\min_{x\in H_{1}, y\in H_{2}}h(x,y), $$

where $\alpha_{i}>0$ $(1\leq i\leq p)$, $\beta_{j}>0$ $(1\leq j\leq r)$, $\sum_{i=1}^{p}\alpha_{i}=1$, and $\sum_{j=1}^{r} \beta_{j}=1$. Assume that the solution set of the MSEP is nonempty, by the optimality conditions $(x^{\ast},y^{\ast})$ solves the MSEP if and only if

2.6 $$ \textstyle\begin{cases} \nabla_{x}h(x^{\ast},y^{\ast})=0,\\ \nabla_{y}h(x^{\ast},y^{\ast})=0, \end{cases} $$

which is equivalent to

2.7 $$ \textstyle\begin{cases} x^{\ast}=x^{\ast}-\gamma\nabla_{x}h(x^{\ast},y^{\ast})=x^{\ast}-\gamma[x^{\ast}-\sum_{i=1}^{p}\alpha_{i}P_{C_{i}}(x^{\ast})+A^{\ast}(Ax^{\ast}-By^{\ast})],\\ y^{\ast}=y^{\ast}-\beta \nabla_{y}h(x^{\ast},y^{\ast})=y^{\ast}-\beta[y^{\ast}-\sum_{j=1}^{r}\beta_{j}P_{Q_{j}}(y^{\ast})-B^{\ast}(Ax^{\ast}-By^{\ast})] \end{cases} $$

for *γ*, $\beta>0$. These motivate us to introduce several iterative algorithms with self-adaptive step size for solving MSECFP (1.1) governed by firmly quasi-nonexpansive mappings and MSEP (1.2).

### The well-known lemmas

The following lemmas will be helpful for our main results in the next section.

#### Lemma 2.1

*Let* *H* *be a real Hilbert space*. *Then*

2.8 $$ 2\langle x,y\rangle= \Vert x \Vert ^{2}+ \Vert y \Vert ^{2}- \Vert x-y \Vert ^{2}= \Vert x+y \Vert ^{2}- \Vert x \Vert ^{2}- \Vert y \Vert ^{2},\quad \forall x,y\in H. $$

#### Lemma 2.2

([36])

*Let* *H* *be a real Hilbert space*. *Then*, *for all* $t\in [0,1]$ *and* $x,y\in H$,

$$\bigl\Vert tx+(1-t)y \bigr\Vert ^{2}=t \Vert x \Vert ^{2}+(1-t) \Vert y \Vert ^{2}-t(1-t) \Vert x-y \Vert ^{2}. $$

#### Lemma 2.3

([37])

*Let* *H* *be a real Hilbert space*. *Then*

$$\Vert \alpha_{0}x_{0}+\alpha_{1}x_{1}+ \alpha_{2}x_{2}+\alpha_{3}x_{3}+\cdots+ \alpha_{r}x_{r} \Vert ^{2} \leq \sum_{i=0}^{r}\alpha_{i} \Vert x_{i} \Vert ^{2}-\alpha_{s}\alpha_{t} \Vert x_{s}-x_{t} \Vert ^{2} $$

*for any* $s,t\in \{0,1,2,\ldots,r\}$ *and for* $x_{i}\in H, i=0,1,2,\ldots,r$, *with* $\alpha_{0}+\alpha_{1}+\cdots+\alpha_{r}=1$ *and* $0\leq\alpha_{i}\leq1$.

#### Lemma 2.4

([38])

*Let* *E* *be a uniformly convex Banach space*, *K* *be a nonempty closed convex subset of* *E*, *and* $T:K\rightarrow K$ *be a nonexpansive mapping*. *Then* $I-T$ *is demi*-*closed at origin*.

## Parallel and cyclic iterative algorithms

In this section, we introduce parallel and cyclic iterative algorithms and prove the weak convergence for solving MSECFP (1.1) of firmly quasi-nonexpansive operators. In our algorithms, the selection of the step size does not need any prior information of the operator norms $\Vert A \Vert $ and $\Vert B \Vert $.

In what follows, we adopt the following assumptions:

1. The problem is consistent, namely its solution set Γ is nonempty;
2. Both $U_{i}$ and $T_{j}$ are firmly quasi-nonexpansive operators, and both $I-U_{i}$ and $I-T_{j}$ are demiclosed at origin ($1\leq i\leq p$, $1\leq j\leq r$).
3. The sequences $\{\alpha_{n}^{i}\}_{i=1}^{p}$, $\{\beta_{n}^{j}\}_{j=1}^{r}\subset[0,1]$ such that $\sum_{i=1}^{p}\alpha_{n}^{i}=1$ and $\sum_{j=1}^{r}\beta_{n}^{j}=1$ for every $n\geq0$, $j(n)=n(\operatorname {mod}r)+1$, $i(n)=n(\operatorname {mod}p)+1$.

### Algorithm 3.1

Let $x_{0}\in H_{1}, y_{0}\in H_{2}$ be arbitrary. For $n\geq 0$, let

3.1 $$ \textstyle\begin{cases} u_{n}=x_{n}-(\alpha _{n}^{1}U_{1}(x_{n})+\cdots+\alpha _{n}^{p}U_{p}(x_{n}))+A^{\ast}(Ax_{n}-By_{n}),\\ x_{n+1}=x_{n}-\tau_{n}u_{n},\\ v_{n}=y_{n}-(\beta _{n}^{1}T_{1}(y_{n})+\cdots+\beta_{n}^{r}T_{r}(y_{n}))-B^{\ast}(Ax_{n}-By_{n}),\\ y_{n+1}=y_{n}-\tau_{n}v_{n}, \end{cases} $$

where the step size $\tau_{n}$ is chosen as

3.2 $$ \tau_{n}\in \biggl(\epsilon,\min \biggl\{ 1, \frac{ \Vert Ax_{n}-By_{n} \Vert ^{2}}{ \Vert A^{\ast}(Ax_{n}-By_{n}) \Vert ^{2}+ \Vert B^{\ast}(Ax_{n}-By_{n}) \Vert ^{2}} \biggr\} -\epsilon \biggr),\quad n\in \Omega, $$

for small enough $\epsilon>0$, otherwise, $\tau_{n}=\tau\in (0,1)$ (*τ* being any value in $(0,1)$), the set of indexes $\Omega=\{n\in N:Ax_{n}-By_{n}\neq0\}$.

### Remark 3.1

Note that in (3.2) the choice of the step size $\tau_{n}$ is independent of the norms $\Vert A \Vert $ and $\Vert B \Vert $. The value of *τ* does not influence the considered algorithm, it was introduced just for the sake of clarity.

### Lemma 3.1

$\tau_{n}$ *defined by* (3.2) *is well defined*.

### Proof

Taking $(x,y)\in \Gamma$, i.e., $x\in \cap_{i=1}^{p}F(U_{i})$, $y\in \cap_{j=1}^{r}F(T_{j})$, and $Ax=By$, we have

$$\bigl\langle A^{\ast}(Ax_{n}-By_{n}), x_{n}-x \bigr\rangle =\langle Ax_{n}-By_{n}, Ax_{n}-Ax\rangle $$

and

$$\bigl\langle B^{\ast}(Ax_{n}-By_{n}), y-y_{n} \bigr\rangle =\langle Ax_{n}-By_{n}, By-By_{n}\rangle. $$

By adding the two above equalities and by taking into account the fact that $Ax = By$, we obtain

3.3 $$ \begin{aligned}[b] & \Vert Ax_{n}-By_{n} \Vert ^{2} \\ &\quad = \bigl\langle A^{\ast}(Ax_{n}-By_{n}), x_{n}-x \bigr\rangle + \bigl\langle B^{\ast}(Ax_{n}-By_{n}), y-y_{n} \bigr\rangle \\ &\quad \leq \bigl\Vert A^{\ast}(Ax_{n}-By_{n}) \bigr\Vert \cdot \Vert x_{n}-x \Vert + \bigl\Vert B^{\ast}(Ax_{n}-By_{n}) \bigr\Vert \cdot \Vert y-y_{n} \Vert . \end{aligned} $$

Consequently, for $n\in \Omega$, that is, $\Vert Ax_{n}-By_{n} \Vert >0$, we have $\Vert A^{\ast}(Ax_{n}-By_{n}) \Vert \neq 0$ or $\Vert B^{\ast}(Ax_{n}-By_{n}) \Vert \neq 0$. This leads to the fact that $\tau_{n}$ is well defined. □

### Theorem 3.1

*Assume that* $\liminf_{n\rightarrow\infty}\alpha_{n}^{i}>0 (1\leq i \leq p)$ *and* $\liminf_{n\rightarrow\infty}\beta_{n}^{j}>0 (1\leq j \leq r)$. *Then the sequence* $\{(x_{n}, y_{n})\}$ *generated by Algorithm *3.1 *weakly converges to a solution* $(x^{\ast},y^{\ast})$ *of MSECFP* (1.1). *Moreover*, $\Vert Ax_{n}-By_{n} \Vert \rightarrow 0$, $\Vert x_{n+1}-x_{n} \Vert \rightarrow0$, *and* $\Vert y_{n+1}-y_{n} \Vert \rightarrow0$ *as* $n\rightarrow\infty$.

### Proof

From the condition on $\{\tau_{n}\}$, we have $\{\tau_{n}\}_{n\geq0}$ is bounded. It follows from Algorithm 3.1 and $\sum_{i=1}^{p}\alpha_{n}^{i}=1$ that

3.4 $$ u_{n}=\alpha_{n}^{1} \bigl(x_{n}-U_{1}(x_{n}) \bigr)+\cdots+ \alpha_{n}^{p} \bigl(x_{n}-U_{p}(x_{n}) \bigr)+A^{\ast}(Ax_{n}-By_{n}). $$

Taking $(x^{\ast},y^{\ast})\in \Gamma$, i.e., $x^{\ast}\in \bigcap_{i=1}^{p}F(U_{i})$, $y^{\ast}\in \bigcap_{j=1}^{r}F(T_{j})$, and $Ax^{\ast}=By^{\ast}$, we have

3.5 $$ \begin{aligned}[b] & \bigl\langle u_{n},x_{n}-x^{\ast} \bigr\rangle \\ &\quad =\alpha_{n}^{1} \bigl\langle x_{n}-U_{1}(x_{n}),x_{n}-x^{\ast} \bigr\rangle +\cdots+\alpha_{n}^{p} \bigl\langle x_{n}-U_{p}(x_{n}),x_{n}-x^{\ast} \bigr\rangle \\ &\qquad {}+ \bigl\langle A^{\ast}(Ax_{n}-By_{n}),x_{n}-x^{\ast} \bigr\rangle \\ &\quad \geq\alpha_{n}^{1} \bigl\Vert x_{n}-U_{1}(x_{n}) \bigr\Vert ^{2}+ \cdots+\alpha_{n}^{p} \bigl\Vert x_{n}-U_{p}(x_{n}) \bigr\Vert ^{2}+ \bigl\langle Ax_{n}-By_{n},Ax_{n}-Ax^{\ast} \bigr\rangle . \end{aligned} $$

Similarly, we have

3.6 $$ \begin{aligned}[b] \bigl\langle v_{n},y_{n}-y^{\ast} \bigr\rangle \geq {}&\beta_{n}^{1} \bigl\Vert y_{n}-T_{1}(y_{n}) \bigr\Vert ^{2}+ \cdots+\beta_{n}^{r} \bigl\Vert y_{n}-T_{r}(y_{n}) \bigr\Vert ^{2} \\ &{}- \bigl\langle Ax_{n}-By_{n},By_{n}-By^{\ast} \bigr\rangle . \end{aligned} $$

By adding the two inequalities (3.5)–(3.6) and taking into account the fact that $Ax^{\ast}= By^{\ast}$, we obtain

3.7 $$ \begin{aligned}[b] & \bigl\langle u_{n},x_{n}-x^{\ast} \bigr\rangle + \bigl\langle v_{n},y_{n}-y^{\ast} \bigr\rangle \\ &\quad \geq\sum_{i=1}^{p} \alpha_{n}^{i} \bigl\Vert x_{n}-U_{i}(x_{n}) \bigr\Vert ^{2}+\sum_{j=1}^{r} \beta_{n}^{j} \bigl\Vert y_{n}-T_{j}(y_{n}) \bigr\Vert ^{2}+ \Vert Ax_{n}-By_{n} \Vert ^{2}. \end{aligned} $$

From Algorithm 3.1 we also have

3.8 $$ \begin{aligned}[b] & \bigl\Vert x_{n+1}-x^{\ast} \bigr\Vert ^{2}+ \bigl\Vert y_{n+1}-y^{\ast} \bigr\Vert ^{2} \\ &\quad = \bigl\Vert x_{n}-\tau_{n}u_{n}-x^{\ast} \bigr\Vert ^{2}+ \bigl\Vert y_{n}-\tau_{n}v_{n}-y^{\ast} \bigr\Vert ^{2} \\ &\quad = \bigl\Vert x_{n}-x^{\ast} \bigr\Vert ^{2}+ \bigl\Vert y_{n}-y^{\ast} \bigr\Vert ^{2}-2\tau_{n} \bigl( \bigl\langle u_{n},x_{n}-x^{\ast} \bigr\rangle + \bigl\langle v_{n},y_{n}-y^{\ast} \bigr\rangle \bigr)+\tau_{n}^{2} \bigl( \Vert u_{n} \Vert ^{2}+ \Vert v_{n} \Vert ^{2} \bigr). \end{aligned} $$

By Lemma 2.3 we get

3.9 $$ \begin{aligned}[b] \Vert u_{n} \Vert ^{2} &= \bigl\Vert \alpha_{n}^{1} \bigl(x_{n}-U_{1}(x_{n}) \bigr)+\cdots+ \alpha_{n}^{p} \bigl(x_{n}-U_{p}(x_{n}) \bigr)+A^{\ast}(Ax_{n}-By_{n}) \bigr\Vert ^{2} \\ &\leq2 \bigl\Vert \alpha_{n}^{1} \bigl(x_{n}-U_{1}(x_{n}) \bigr)+\cdots+ \alpha_{n}^{p} \bigl(x_{n}-U_{p}(x_{n}) \bigr) \bigr\Vert ^{2}+2 \bigl\Vert A^{\ast}(Ax_{n}-By_{n}) \bigr\Vert ^{2} \\ &\leq2\sum_{i=1}^{p} \alpha_{n}^{i} \bigl\Vert x_{n}-U_{i}(x_{n}) \bigr\Vert ^{2}+2 \bigl\Vert A^{\ast}(Ax_{n}-By_{n}) \bigr\Vert ^{2} \end{aligned} $$

and

3.10 $$ \Vert v_{n} \Vert ^{2}\leq2\sum _{j=1}^{r}\beta_{n}^{j} \bigl\Vert y_{n}-T_{j}(y_{n}) \bigr\Vert ^{2}+2 \bigl\Vert B^{\ast}(Ax_{n}-By_{n}) \bigr\Vert ^{2}. $$

Setting $s_{n}(x^{\ast},y^{\ast})= \Vert x_{n}-x^{\ast} \Vert ^{2}+ \Vert y_{n}-y^{\ast} \Vert ^{2}$ and using (3.7), (3.9)–(3.10), (3.8) can be written as

3.11 $$\begin{aligned} &s_{n+1} \bigl(x^{\ast},y^{\ast} \bigr) \\ &\quad \leq s_{n} \bigl(x^{\ast},y^{\ast} \bigr) \\ &\qquad {}-2\tau_{n} \Biggl[\sum_{i=1}^{p} \alpha_{n}^{i} \bigl\Vert x_{n}-U_{i}(x_{n}) \bigr\Vert ^{2}+\sum_{j=1}^{r} \beta_{n}^{j} \bigl\Vert y_{n}-T_{j}(y_{n}) \bigr\Vert ^{2}+ \Vert Ax_{n}-By_{n} \Vert ^{2} \Biggr] \\ &\qquad {}+2\tau_{n}^{2} \Biggl[\sum _{i=1}^{p}\alpha_{n}^{i} \bigl\Vert x_{n}-U_{i}(x_{n}) \bigr\Vert ^{2}+\sum_{j=1}^{r} \beta_{n}^{j} \bigl\Vert y_{n}-T_{j}(y_{n}) \bigr\Vert ^{2} \\ &\qquad {}+ \bigl\Vert A^{\ast}(Ax_{n}-By_{n}) \bigr\Vert ^{2}+ \bigl\Vert B^{\ast}(Ax_{n}-By_{n}) \bigr\Vert ^{2} \Biggr] \\ &\quad =s_{n} \bigl(x^{\ast},y^{\ast} \bigr)-2 \tau_{n}(1-\tau_{n}) \Biggl[\sum _{i=1}^{p}\alpha_{n}^{i} \bigl\Vert x_{n}-U_{i}(x_{n}) \bigr\Vert ^{2}+\sum_{j=1}^{r} \beta_{n}^{j} \bigl\Vert y_{n}-T_{j}(y_{n}) \bigr\Vert ^{2} \Biggr] \\ &\qquad {}-2\tau_{n} \bigl[ \Vert Ax_{n}-By_{n} \Vert ^{2}-\tau_{n} \bigl( \bigl\Vert A^{\ast}(Ax_{n}-By_{n}) \bigr\Vert ^{2}+ \bigl\Vert B^{\ast}(Ax_{n}-By_{n}) \bigr\Vert ^{2} \bigr) \bigr]. \end{aligned}$$

We see that the sequence $\{s_{n}(x^{\ast},y^{\ast})\}$ is decreasing and lower bounded by 0; consequently, it converges to some finite limit which is denoted by $s(x^{\ast},y^{\ast})$. So the sequences $\{x_{n}\}$ and $\{y_{n}\}$ are bounded.

By the conditions on $\{\tau_{n}\}$, $\{\alpha_{n}^{i}\}$ ($1\leq i\leq p$) and $\{\beta_{n}^{j}\}$ ($1\leq j\leq r$), from (3.11) we obtain, for all *i* ($1\leq i\leq p$) and *j* ($1\leq j\leq r$),

3.12 $$ \lim_{n\rightarrow\infty} \bigl\Vert x_{n}-U_{i}(x_{n}) \bigr\Vert =\lim_{n\rightarrow\infty} \bigl\Vert y_{n}-T_{j}(y_{n}) \bigr\Vert =0 $$

and

3.13 $$ \lim_{n\rightarrow\infty} \bigl\Vert A^{\ast}(Ax_{n}-By_{n}) \bigr\Vert =\lim_{n\rightarrow\infty} \bigl\Vert B^{\ast}(Ax_{n}-By_{n}) \bigr\Vert =0. $$

It follows from (3.3) and (3.13) that

3.14 $$ \lim_{n\rightarrow\infty} \Vert Ax_{n}-By_{n} \Vert =0. $$

Since

3.15 $$ \begin{aligned}[b] & \Vert x_{n+1}-x_{n} \Vert \\ &\quad = \Vert x_{n}-\tau_{n}u_{n}-x_{n} \Vert \\ &\quad =\tau_{n} \bigl\Vert \alpha_{n}^{1} \bigl(x_{n}-U_{1}(x_{n}) \bigr)+\cdots+ \alpha_{n}^{p} \bigl(x_{n}-U_{p}(x_{n}) \bigr)+A^{\ast}(Ax_{n}-By_{n}) \bigr\Vert \\ &\quad \leq\tau_{n} \bigl(\alpha_{n}^{1} \bigl\Vert x_{n}-U_{1}(x_{n}) \bigr\Vert +\cdots+ \alpha_{n}^{p} \bigl\Vert x_{n}-U_{p}(x_{n}) \bigr\Vert + \bigl\Vert A^{\ast}(Ax_{n}-By_{n}) \bigr\Vert \bigr), \end{aligned} $$

we get

3.16 $$ \lim_{n\rightarrow\infty} \Vert x_{n+1}-x_{n} \Vert =0, $$

which infers that $\{x_{n}\}$ is asymptotically regular. Similarly, we also have that $\{y_{n}\}$ is asymptotically regular, namely $\lim_{n\rightarrow\infty} \Vert y_{n+1}-y_{n} \Vert =0$.

Take $(\tilde{x},\tilde{y})\in \omega_{\omega}(x_{n},y_{n})$, i.e., there exists a subsequence $\{(x_{n_{k}},y_{n_{k}})\}$ of $\{(x_{n},y_{n})\}$ such that $(x_{n_{k}},y_{n_{k}})\rightharpoonup(\tilde{x},\tilde{y})$ as $k\rightarrow\infty$. Combined with the demiclosedness of $U_{i}-I$ and $T_{j}-I$ at 0, it follows from (3.12) that $U_{i}(\tilde{x})=\tilde{x}$ and $T_{j}(\tilde{y})=\tilde{y}$ for $1\leq i\leq p$ and $1\leq j\leq r$. So, $\tilde{x}\in \bigcap_{i=1}^{p}F(U_{i})$ and $\tilde{y}\in \bigcap_{j=1}^{r}F(T_{j})$. On the other hand, $A\tilde{x}-B\tilde{y}\in\omega_{w}(Ax_{n}-By_{n})$ and weakly lower semicontinuity of the norm imply that

$$\Vert A\tilde{x}-B\tilde{y} \Vert \leq\liminf_{n\rightarrow\infty} \Vert Ax_{n}-By_{n} \Vert =0, $$

hence $(\tilde{x},\tilde{y})\in \Gamma$. So $\omega_{w}(x_{n},y_{n})\subseteq \Gamma$.

Next, we will show the uniqueness of the weak cluster point $\{(x_{n},y_{n})\}$. Indeed, let $(\bar{x},\bar{y})$ be another weak cluster point of $\{(x_{n},y_{n})\}$, then $(\bar{x},\bar{y})\in \Gamma$. From the definition of $s_{n}(x^{\ast},y^{\ast})$, we have

3.17 $$\begin{aligned} s_{n}(\tilde{x},\tilde{y}) = &\Vert x_{n}-\bar{x} \Vert ^{2}+ \Vert \bar{x}-\tilde{x} \Vert ^{2}+2\langle x_{n}-\bar{x}, \bar{x}-\tilde{x} \rangle+ \Vert y_{n}-\bar{y} \Vert ^{2}+ \Vert \bar{y}- \tilde{y} \Vert ^{2}+2\langle y_{n}- \bar{y},\bar{y}- \tilde{y}\rangle \\ =&s_{n}(\bar{x},\bar{y})+ \Vert \bar{x}-\tilde{x} \Vert ^{2}+ \Vert \bar{y}-\tilde{y} \Vert ^{2}+2\langle x_{n}-\bar{x},\bar{x}-\tilde{x}\rangle+2\langle y_{n}- \bar{y},\bar{y}-\tilde{y}\rangle. \end{aligned}$$

Without loss of generality, we may assume that $x_{n}\rightharpoonup \bar{x}$ and $y_{n}\rightharpoonup \bar{y}$. By passing to the limit in relation (3.17), we obtain

$$s(\tilde{x},\tilde{y})=s(\bar{x},\bar{y})+ \Vert \bar{x}-\tilde{x} \Vert ^{2}+ \Vert \bar{y}-\tilde{y} \Vert ^{2}. $$

Reversing the role of $(\tilde{x},\tilde{y})$ and $(\bar{x},\bar{y})$, we also have

$$s(\bar{x},\bar{y})=s(\tilde{x},\tilde{y})+ \Vert \tilde{x}-\bar{x} \Vert ^{2}+ \Vert \tilde{y}-\bar{y} \Vert ^{2}. $$

By adding the two last equalities, we obtain $\tilde{x}=\bar{x}$ and $\tilde{y}=\bar{y}$, which implies that $\{(x_{n}, y_{n})\}$ weakly converges to the solution of (1.1). This completes the proof. □

Next, we propose the cyclic iterative algorithm for solving MSECFP (1.1) of firmly quasi-nonexpansive operators.

### Algorithm 3.2

Let $x_{0}\in H_{1}, y_{0}\in H_{2}$ be arbitrary. For $n\geq 0$, let

3.18 $$ \textstyle\begin{cases} u_{n}=x_{n}-U_{i(n)}(x_{n})+A^{\ast}(Ax_{n}-By_{n}),\\ x_{n+1}=x_{n}-\tau_{n}u_{n},\\ v_{n}=y_{n}-T_{j(n)}(y_{n})-B^{\ast}(Ax_{n}-By_{n}),\\ y_{n+1}=y_{n}-\tau_{n}v_{n}, \end{cases} $$

where the step size $\tau_{n}$ is chosen as in Algorithm 3.1.

### Theorem 3.2

*The sequence* $\{(x_{n}, y_{n})\}$ *generated by Algorithm *3.2 *weakly converges to a solution* $(x^{\ast},y^{\ast})$ *of MSECFP* (1.1). *Moreover*, $\Vert Ax_{n}-By_{n} \Vert \rightarrow 0$, $\Vert x_{n}-x_{n+1} \Vert \rightarrow0$, *and* $\Vert y_{n}-y_{n+1} \Vert \rightarrow0$ *as* $n\rightarrow\infty$.

### Proof

Let $(x^{\ast},y^{\ast})\in\Gamma$, we have

3.19 $$ \begin{aligned}[b] \bigl\langle u_{n},x_{n}-x^{\ast} \bigr\rangle &= \bigl\langle x_{n}-U_{i(n)}(x_{n}),x_{n}-x^{\ast} \bigr\rangle + \bigl\langle A^{\ast}(Ax_{n}-By_{n}),x_{n}-x^{\ast} \bigr\rangle \\ &\geq \bigl\Vert x_{n}-U_{i(n)}(x_{n}) \bigr\Vert ^{2}+ \bigl\langle Ax_{n}-By_{n},Ax_{n}-Ax^{\ast} \bigr\rangle \end{aligned} $$

and

3.20 $$ \bigl\langle v_{n},y_{n}-y^{\ast} \bigr\rangle \geq \bigl\Vert y_{n}-T_{j(n)}(y_{n}) \bigr\Vert ^{2}- \bigl\langle Ax_{n}-By_{n},By_{n}-By^{\ast} \bigr\rangle . $$

By adding the two inequalities (3.19)–(3.20) and taking into account the fact that $Ax^{\ast}= By^{\ast}$, we obtain

3.21 $$ \begin{aligned}[b] & \bigl\langle u_{n},x_{n}-x^{\ast} \bigr\rangle + \bigl\langle v_{n},y_{n}-y^{\ast} \bigr\rangle \\ &\quad \geq \bigl\Vert x_{n}-U_{i(n)}(x_{n}) \bigr\Vert ^{2}+ \bigl\Vert y_{n}-T_{j(n)}(y_{n}) \bigr\Vert ^{2}+ \Vert Ax_{n}-By_{n} \Vert ^{2}. \end{aligned} $$

Similar to (3.8), we have

3.22 $$ \begin{aligned}[b] & \bigl\Vert x_{n+1}-x^{\ast} \bigr\Vert ^{2}+ \bigl\Vert y_{n+1}-y^{\ast} \bigr\Vert ^{2} \\ &\quad = \bigl\Vert x_{n}-x^{\ast} \bigr\Vert ^{2}+ \bigl\Vert y_{n}-y^{\ast} \bigr\Vert ^{2}-2 \tau_{n} \bigl( \bigl\langle u_{n},x_{n}-x^{\ast} \bigr\rangle + \bigl\langle v_{n},y_{n}-y^{\ast} \bigr\rangle \bigr) \\ &\qquad {}+\tau_{n}^{2} \bigl( \Vert u_{n} \Vert ^{2}+ \Vert v_{n} \Vert ^{2} \bigr). \end{aligned} $$

We also have

3.23 $$ \begin{aligned}[b] \Vert u_{n} \Vert ^{2}&= \bigl\Vert x_{n}-U_{i(n)}(x_{n})+A^{\ast}(Ax_{n}-By_{n}) \bigr\Vert ^{2} \\ &\leq2 \bigl\Vert x_{n}-U_{i(n)}(x_{n}) \bigr\Vert ^{2}+2 \bigl\Vert A^{\ast}(Ax_{n}-By_{n}) \bigr\Vert ^{2} \end{aligned} $$

and

3.24 $$ \Vert v_{n} \Vert ^{2} \leq2 \bigl\Vert y_{n}-T_{j(n)}(y_{n}) \bigr\Vert ^{2}+2 \bigl\Vert B^{\ast}(Ax_{n}-By_{n}) \bigr\Vert ^{2}. $$

Setting $s_{n}(x^{\ast},y^{\ast})= \Vert x_{n}-x^{\ast} \Vert ^{2}+ \Vert y_{n}-y^{\ast} \Vert ^{2}$ and using (3.21), (3.23)–(3.24), (3.22) can be written as

3.25 $$ \begin{aligned}[b] &s_{n+1} \bigl(x^{\ast},y^{\ast} \bigr) \\ &\quad \leq s_{n} \bigl(x^{\ast},y^{\ast} \bigr)-2 \tau_{n} \bigl[ \bigl\Vert x_{n}-U_{i(n)}(x_{n}) \bigr\Vert ^{2}+ \bigl\Vert y_{n}-T_{j(n)}(y_{n}) \bigr\Vert ^{2}+ \Vert Ax_{n}-By_{n} \Vert ^{2} \bigr] \\ &\qquad {}+2\tau_{n}^{2} \bigl[ \bigl\Vert x_{n}-U_{i(n)}(x_{n}) \bigr\Vert ^{2}+ \bigl\Vert y_{n}-T_{j(n)}(y_{n}) \bigr\Vert ^{2}+ \bigl\Vert A^{\ast}(Ax_{n}-By_{n}) \bigr\Vert ^{2} \\ &\qquad {}+ \bigl\Vert B^{\ast}(Ax_{n}-By_{n}) \bigr\Vert ^{2} \bigr] \\ &\quad =s_{n} \bigl(x^{\ast},y^{\ast} \bigr)-2 \tau_{n}(1-\tau_{n}) \bigl[ \bigl\Vert x_{n}-U_{i(n)}(x_{n}) \bigr\Vert ^{2}+ \bigl\Vert y_{n}-T_{j(n)}(y_{n}) \bigr\Vert ^{2} \bigr] \\ &\qquad {}-2\tau_{n} \bigl[ \Vert Ax_{n}-By_{n} \Vert ^{2}-\tau_{n} \bigl( \bigl\Vert A^{\ast}(Ax_{n}-By_{n}) \bigr\Vert ^{2}+ \bigl\Vert B^{\ast}(Ax_{n}-By_{n}) \bigr\Vert ^{2} \bigr) \bigr]. \end{aligned} $$

Similar to the proof of Theorem 3.1, we have

3.26 $$ \lim_{n\rightarrow\infty} \bigl\Vert x_{n}-U_{i(n)}(x_{n}) \bigr\Vert =\lim_{n\rightarrow\infty} \bigl\Vert y_{n}-T_{j(n)}(y_{n}) \bigr\Vert =0 $$

and

3.27 $$ \lim_{n\rightarrow\infty} \Vert Ax_{n}-By_{n} \Vert =0. $$

Since

3.28 $$ \begin{aligned}[b] \Vert x_{n+1}-x_{n} \Vert &=\tau_{n} \bigl\Vert x_{n}-U_{i(n)}(x_{n})+A^{\ast}(Ax_{n}-By_{n}) \bigr\Vert \\ &\leq \tau_{n} \bigl( \bigl\Vert x_{n}-U_{i(n)}(x_{n}) \bigr\Vert + \bigl\Vert A^{\ast}(Ax_{n}-By_{n}) \bigr\Vert \bigr), \end{aligned} $$

we get

3.29 $$ \lim_{n\rightarrow\infty} \Vert x_{n+1}-x_{n} \Vert =0, $$

which infers that $\{x_{n}\}$ is asymptotically regular. Similarly, we also have that $\{y_{n}\}$ is asymptotically regular, namely $\lim_{n\rightarrow\infty} \Vert y_{n+1}-y_{n} \Vert =0$.

Take $(\tilde{x},\tilde{y})\in \omega_{\omega}(x_{n},y_{n})$, i.e., there exists a subsequence $\{(x_{n_{k}},y_{n_{k}})\}$ of $\{(x_{n},y_{n})\}$ such that $(x_{n_{k}},y_{n_{k}})\rightharpoonup(\tilde{x},\tilde{y})$ as $k\rightarrow\infty$. Noting that the pool of indexes is finite and $\{x_{n}\}$ is asymptotically regular, for any $i\in \{1,2,\ldots,p\}$, we can choose a subsequence $\{n_{i_{l}}\}\subset\{n\}$ such that $x_{n_{i_{l}}}\rightharpoonup \tilde{x}$ as $l\rightarrow\infty$ and $i(n_{i_{l}})=i$ for all *l*. It turns out that

3.30 $$ \lim_{l\rightarrow\infty} \bigl\Vert x_{n_{i_{l}}}-U_{i}(x_{n_{i_{l}}}) \bigr\Vert =\lim_{l\rightarrow\infty} \bigl\Vert x_{n_{i_{l}}}-U_{i(n_{i_{l}})}(x_{n_{i_{l}}}) \bigr\Vert =0. $$

By the same reason, for any $j\in \{1,2,\ldots,r\}$, we can choose a subsequence $\{n_{j_{m}}\}\subset\{n\}$ such that $y_{n_{j_{m}}}\rightharpoonup \tilde{y}$ as $m\rightarrow\infty$ and $j(n_{j_{m}})=j$ for all *m*. So,

3.31 $$ \lim_{m\rightarrow\infty} \bigl\Vert y_{n_{j_{m}}}-U_{j}(y_{n_{j_{m}}}) \bigr\Vert =0. $$

Combined with the demiclosedness of $U_{i}-I$ and $T_{j}-I$ at 0, it follows from (3.30) and (3.31) that $U_{i}(\tilde{x})=\tilde{x}$ and $T_{j}(\tilde{y})=\tilde{y}$ for $1\leq i\leq p$ and $1\leq j\leq r$. So, $\tilde{x}\in \bigcap_{i=1}^{p}F(U_{i})$ and $\tilde{y}\in \bigcap_{j=1}^{r}F(T_{j})$. Similar to the proof of Theorem 3.1, we can complete the proof. □

Now, we give applications of Theorem 3.1 and Theorem 3.2 to solve MSEP (1.2). Assume that the solution set *S* of MSEP (1.2) is nonempty. Since the orthogonal projection operator is firmly nonexpansive, by Lemma 2.4 we have the following results for solving MSEP (1.2).

### Corollary 3.1

*For any given* $x_{0}\in H_{1}, y_{0}\in H_{2}$, *define a sequence* $\{(x_{n},y_{n})\}$ *by the following procedure*:

3.32 $$ \textstyle\begin{cases} u_{n}=x_{n}-(\alpha _{n}^{1}P_{C_{1}}(x_{n})+\cdots+\alpha _{n}^{p}P_{C_{p}}(x_{n}))+A^{\ast}(Ax_{n}-By_{n}),\\ x_{n+1}=x_{n}-\tau_{n}u_{n},\\ v_{n}=y_{n}-(\beta _{n}^{1}P_{Q_{1}}(y_{n})+\cdots+\beta_{n}^{r}P_{Q_{r}}(y_{n}))-B^{\ast}(Ax_{n}-By_{n}),\\ y_{n+1}=y_{n}-\tau_{n}v_{n}, \end{cases} $$

*where the step size* $\tau_{n}$ *is chosen as in Algorithm *3.1. *If* $\liminf_{n\rightarrow\infty}\alpha_{n}^{i}>0$ ($1\leq i \leq p$) *and* $\liminf_{n\rightarrow\infty}\beta_{n}^{j}>0$ ($1\leq j \leq r$), *then the sequence* $\{(x_{n}, y_{n})\}$ *weakly converges to a solution* $(x^{\ast},y^{\ast})$ *of MSEP* (1.2). *Moreover*, $\Vert Ax_{n}-By_{n} \Vert \rightarrow 0$, $\Vert x_{n+1}-x_{n} \Vert \rightarrow0$, *and* $\Vert y_{n+1}-y_{n} \Vert \rightarrow0$ *as* $n\rightarrow\infty$.

### Corollary 3.2

*For any given* $x_{0}\in H_{1}, y_{0}\in H_{2}$, *define a sequence* $\{(x_{n},y_{n})\}$ *by the following procedure*:

3.33 $$ \textstyle\begin{cases} u_{n}=x_{n}-P_{C_{i(n)}}(x_{n})+A^{\ast}(Ax_{n}-By_{n}),\\ x_{n+1}=x_{n}-\tau_{n}u_{n},\\ v_{n}=y_{n}-P_{Q_{j(n)}}(y_{n})-B^{\ast}(Ax_{n}-By_{n}),\\ y_{n+1}=y_{n}-\tau_{n}v_{n}, \end{cases} $$

*where the step size* $\tau_{n}$ *is chosen as in Algorithm *3.1. *Then the sequence* $\{(x_{n}, y_{n})\}$ *weakly converges to a solution* $(x^{\ast},y^{\ast})$ *of MSEP* (1.2). *Moreover*, $\Vert Ax_{n}-By_{n} \Vert \rightarrow 0$, $\Vert x_{n+1}-x_{n} \Vert \rightarrow0$, *and* $\Vert y_{n+1}-y_{n} \Vert \rightarrow0$ *as* $n\rightarrow\infty$.

## Mixed cyclic and parallel iterative algorithms

Now, for solving MSECFP (1.1) of firmly quasi-nonexpansive operators, we introduce two mixed iterative algorithms which combine the process of cyclic and simultaneous iterative methods. In our algorithms, the selection of the step size does not need any prior information of the operator norms $\Vert A \Vert $ and $\Vert B \Vert $, and the weak convergence is proved. We go on making use of assumptions (A1)–(A3).

### Algorithm 4.1

Let $x_{0}\in H_{1}, y_{0}\in H_{2}$ be arbitrary. For $n\geq 0$, let

4.1 $$ \textstyle\begin{cases} u_{n}=x_{n}-(\alpha _{n}^{1}U_{1}(x_{n})+\cdots+\alpha _{n}^{p}U_{p}(x_{n}))+A^{\ast}(Ax_{n}-By_{n}),\\ x_{n+1}=x_{n}-\tau_{n}u_{n},\\ v_{n}=y_{n}-T_{j(n)}(y_{n})-B^{\ast}(Ax_{n}-By_{n}),\\ y_{n+1}=y_{n}-\tau_{n}v_{n}, \end{cases} $$

where the step size $\tau_{n}$ is chosen in the same way as in Algorithm 3.1.

### Theorem 4.1

*Assume that* $\liminf_{n\rightarrow\infty}\alpha_{n}^{i}>0$ ($1\leq i \leq p$). *Then the sequence* $\{(x_{n}, y_{n})\}$ *generated by Algorithm *4.1 *weakly converges to a solution* $(x^{\ast},y^{\ast})$ *of MSECFP* (1.1). *Moreover*, $\Vert Ax_{n}-By_{n} \Vert \rightarrow 0$, $\Vert x_{n+1}-x_{n} \Vert \rightarrow0$, *and* $\Vert y_{n+1}-y_{n} \Vert \rightarrow0$ *as* $n\rightarrow\infty$.

### Proof

Let $(x^{\ast},y^{\ast})\in \Gamma$. We can get (3.5) and (3.20), so

4.2 $$ \begin{aligned}[b] & \bigl\langle u_{n},x_{n}-x^{\ast} \bigr\rangle + \bigl\langle v_{n},y_{n}-y^{\ast} \bigr\rangle \\ &\quad \geq\sum_{i=1}^{p} \alpha_{n}^{i} \bigl\Vert x_{n}-U_{i}(x_{n}) \bigr\Vert ^{2}+ \bigl\Vert y_{n}-T_{j(n)}(y_{n}) \bigr\Vert ^{2}+ \Vert Ax_{n}-By_{n} \Vert ^{2}. \end{aligned} $$

It follows from Algorithm 4.1 that (3.8)–(3.9) and (3.24) are true. Setting $s_{n}(x^{\ast},y^{\ast})= \Vert x_{n}-x^{\ast} \Vert ^{2}+ \Vert y_{n}-y^{\ast} \Vert ^{2}$, we have

4.3 $$ \begin{aligned}[b] &s_{n+1} \bigl(x^{\ast},y^{\ast} \bigr) \\ &\quad \leq s_{n} \bigl(x^{\ast},y^{\ast} \bigr)-2 \tau_{n}(1-\tau_{n}) \bigl[\alpha_{n}^{1} \bigl\Vert x_{n}-U_{1}(x_{n}) \bigr\Vert ^{2}+\cdots+\alpha_{n}^{p} \bigl\Vert x_{n}-U_{p}(x_{n}) \bigr\Vert ^{2} \\ &\qquad {}+ \bigl\Vert y_{n}-T_{j(n)}(y_{n}) \bigr\Vert ^{2} \bigr] -2\tau_{n} \bigl[ \Vert Ax_{n}-By_{n} \Vert ^{2}-\tau_{n} \bigl( \bigl\Vert A^{\ast}(Ax_{n}-By_{n}) \bigr\Vert ^{2} \\ &\qquad {}+ \bigl\Vert B^{\ast}(Ax_{n}-By_{n}) \bigr\Vert ^{2} \bigr) \bigr]. \end{aligned} $$

By the same reason as in Theorem 3.1, we obtain that, for all *i* $(1\leq i\leq p)$,

4.4 $$\begin{aligned} &\lim_{n\rightarrow\infty} \bigl\Vert x_{n}-U_{i}(x_{n}) \bigr\Vert =0, \end{aligned}$$

4.5 $$\begin{aligned} &\lim_{n\rightarrow\infty} \bigl\Vert y_{n}-T_{j(n)}(y_{n}) \bigr\Vert =0 \end{aligned}$$

and

4.6 $$ \lim_{n\rightarrow\infty} \Vert Ax_{n}-By_{n} \Vert =0. $$

So

4.7 $$ \lim_{n\rightarrow\infty} \Vert x_{n+1}-x_{n} \Vert =\lim_{n\rightarrow\infty} \Vert y_{n+1}-y_{n} \Vert =0, $$

which infers that $\{x_{n}\}$ and $\{y_{n}\}$ are asymptotically regular.

Take $(\tilde{x},\tilde{y})\in \omega_{\omega}(x_{n},y_{n})$, i.e., there exists a subsequence $\{(x_{n_{k}},y_{n_{k}})\}$ of $\{(x_{n},y_{n})\}$ such that $(x_{n_{k}},y_{n_{k}})\rightharpoonup(\tilde{x},\tilde{y})$ as $k\rightarrow\infty$. Noting that the pool of indexes is finite and $\{y_{n}\}$ is asymptotically regular, for any $j\in \{1,2,\ldots,r\}$, we can choose a subsequence $\{n_{j_{l}}\}\subset\{n\}$ such that $y_{n_{j_{l}}}\rightharpoonup \tilde{y}$ as $l\rightarrow\infty$ and $j(n_{j_{l}})=j$ for all *l*. It turns out that

4.8 $$ \lim_{l\rightarrow\infty} \bigl\Vert y_{n_{j_{l}}}-T_{j}(y_{n_{j_{l}}}) \bigr\Vert =\lim_{l\rightarrow\infty} \bigl\Vert y_{n_{j_{l}}}-T_{j(n_{j_{l}})}(y_{n_{j_{l}}}) \bigr\Vert =0. $$

Combined with the demiclosedness of $U_{i}-I$ and $T_{j}-I$ at 0, it follows from (4.4) and (4.7) that $U_{i}(\tilde{x})=\tilde{x}$ and $T_{j}(\tilde{y})=\tilde{y}$ for $1\leq i\leq p$ and $1\leq j\leq r$. So, $\tilde{x}\in \bigcap_{i=1}^{p}F(U_{i})$ and $\tilde{y}\in \bigcap_{j=1}^{r}F(T_{j})$. Similar to the proof of Theorem 3.1, we can complete the proof. □

Next, we propose another mixed cyclic and parallel iterative algorithm for solving MSECFP (1.1) of firmly quasi-nonexpansive operators.

### Algorithm 4.2

Let $x_{0}\in H_{1}, y_{0}\in H_{2}$ be arbitrary. For $n\geq 0$, let

4.9 $$ \textstyle\begin{cases} u_{n}=x_{n}-U_{i(n)}(x_{n})+A^{\ast}(Ax_{n}-By_{n}),\\ x_{n+1}=x_{n}-\tau_{n}u_{n},\\ v_{n}=y_{n}-(\beta_{n}^{1}T_{1}(y_{n})+\cdots+\beta_{n}^{r}T_{r}(y_{n}))-B^{\ast}(Ax_{n}-By_{n}),\\ y_{n+1}=y_{n}-\tau_{n}v_{n}, \end{cases} $$

where the step size $\tau_{n}$ is chosen as in Algorithm 3.1.

Similar to the proof of Theorem 4.1, we can get the following result.

### Theorem 4.2

*Assume that* $\liminf_{n\rightarrow\infty}\beta_{n}^{j}>0$ ($1\leq j \leq r$). *Then the sequence* $\{(x_{n}, y_{n})\}$ *generated by Algorithm *4.2 *weakly converges to a solution* $(x^{\ast},y^{\ast})$ *of MSECFP* (1.1) *of firmly quasi*-*nonexpansive operators*. *Moreover*, $\Vert Ax_{n}-By_{n} \Vert \rightarrow 0$, $\Vert x_{n}-x_{n+1} \Vert \rightarrow0$ *and* $\Vert y_{n}-y_{n+1} \Vert \rightarrow0$ *as* $n\rightarrow\infty$.

Finally, we obtain two mixed iterative algorithms to solve MSEP (1.2). Assume that the solution set *S* of MSEP (1.2) is nonempty.

### Corollary 4.1

*For any given* $x_{0}\in H_{1}, y_{0}\in H_{2}$, *define a sequence* $\{(x_{n},y_{n})\}$ *by the following procedure*:

4.10 $$ \textstyle\begin{cases} u_{n}=x_{n}-(\alpha _{n}^{1}P_{C_{1}}(x_{n})+\cdots+\alpha _{n}^{p}P_{C_{p}}(x_{n}))+A^{\ast}(Ax_{n}-By_{n}),\\ x_{n+1}=x_{n}-\tau_{n}u_{n},\\ v_{n}=y_{n}-P_{Q_{j(n)}}(y_{n})-B^{\ast}(Ax_{n}-By_{n}),\\ y_{n+1}=y_{n}-\tau_{n}v_{n}, \end{cases} $$

*where the step size* $\tau_{n}$ *is chosen as in Algorithm *3.1. *If* $\liminf_{n\rightarrow\infty}\alpha_{n}^{i}>0$ ($1\leq i \leq p$), *then the sequence* $\{(x_{n}, y_{n})\}$ *weakly converges to a solution* $(x^{\ast},y^{\ast})$ *of MSEP* (1.2). *Moreover*, $\Vert Ax_{n}-By_{n} \Vert \rightarrow 0$, $\Vert x_{n+1}-x_{n} \Vert \rightarrow0$, *and* $\Vert y_{n+1}-y_{n} \Vert \rightarrow0$ *as* $n\rightarrow\infty$.

### Corollary 4.2

*For any given* $x_{0}\in H_{1}$, $y_{0}\in H_{2}$, *define a sequence* $\{(x_{n},y_{n})\}$ *by the following procedure*:

4.11 $$ \textstyle\begin{cases} _{n}=x_{n}-P_{C_{i(n)}}(x_{n})+A^{\ast}(Ax_{n}-By_{n}),\\ x_{n+1}=x_{n}-\tau_{n}u_{n},\\ v_{n}=y_{n}-(\beta_{n}^{1}P_{Q_{1}}(y_{n})+\cdots+\beta_{n}^{r}P_{Q_{r}}(y_{n}))-B^{\ast}(Ax_{n}-By_{n}),\\ y_{n+1}=y_{n}-\tau_{n}v_{n}, \end{cases} $$

*where the step size* $\tau_{n}$ *is chosen as in Algorithm *3.1. *If* $\liminf_{n\rightarrow\infty}\beta_{n}^{j}>0 (1\leq j \leq r)$, *then the sequence* $\{(x_{n}, y_{n})\}$ *weakly converges to a solution* $(x^{\ast},y^{\ast})$ *of MSEP* (1.2). *Moreover*, $\Vert Ax_{n}-By_{n} \Vert \rightarrow 0$, $\Vert x_{n+1}-x_{n} \Vert \rightarrow0$, *and* $\Vert y_{n+1}-y_{n} \Vert \rightarrow0$ *as* $n\rightarrow\infty$.

## Results and discussion

To avoid computing the norms of the bounded linear operators, we introduce parallel and cyclic iterative algorithms with self-adaptive step size to solve MSECFP (1.1) governed by firmly quasi-nonexpansive operators. We also propose two mixed iterative algorithms and do not need the norms of bounded linear operators. As applications, we obtain several iterative algorithms to solve MSEP (1.2).

## Conclusion

In this paper, we have considered MSECFP (1.1) of firmly quasi-nonexpansive operators. Inspired by the methods for solving SCFP (2.1) and MSCFP (1.3), we introduce parallel and cyclic iterative algorithms for solving MSECFP (1.1). We also present two mixed iterative algorithms which combine the process of parallel and cyclic iterative methods. In our several iterative algorithms, the step size is chosen in a self-adaptive way and the weak convergence is proved.

## References

[1] Moudafi A. (2014). Alternating CQ-algorithms for convex feasibility and split fixed-point problems. J. Nonlinear Convex Anal..

[2] Attouch H., Bolte J., Redont P., Soubeyran A. (2008). Alternating proximal algorithms for weakly coupled minimization problems. Applications to dynamical games and PDE’s. J. Convex Anal..

[3] Censor Y., Bortfeld T., Martin B., Trofimov A. (2006). A unified approach for inversion problems in intensity-modulated radiation therapy. Phys. Med. Biol..

[4] Byrne C. (2004). A unified treatment of some iterative algorithms in signal processing and image reconstruction. Inverse Probl..

[5] Censor Y., Elfving T. (1994). A multiprojection algorithm using Bregman projections in a product space. Numer. Algorithms.

[6] Censor Y., Elfving T., Kopf N., Bortfeld T. (2005). The multiple-sets split feasibility problem and its applications for inverse problems. Inverse Probl..

[7] Censor Y., Gibali A., Reich S. (2012). Algorithms for the split variational inequality problem. Numer. Algorithms.

[8] Qu B., Xiu N. (2005). A note on the CQ algorithm for the split feasibility problem. Inverse Probl..

[9] Xu H.K. (2006). A variable Krasnosel’skiĭ–Mann algorithm and the multiple-set split feasibility problem. Inverse Probl..

[10] Xu H.K. (2010). Iterative methods for the split feasibility problem in infinite-dimensional Hilbert spaces. Inverse Probl..

[11] Yang Q. (2004). The relaxed CQ algorithm solving the split feasibility problem. Inverse Probl..

[12] Masad E., Reich S. (2007). A note on the multiple-set split convex feasibility problem in Hilbert space. J. Nonlinear Convex Anal..

[13] Yao Y., Yao Z., Abdou A.A.N., Cho Y.J. (2015). Self-adaptive algorithms for proximal split feasibility problems and strong convergence analysis. Fixed Point Theory Appl..

[14] Wen M., Peng J., Tang Y. (2015). A cyclic and simultaneous iterative method for solving the multiple-sets split feasibility problem. J. Optim. Theory Appl..

[15] Qin X., Yao J.C. (2017). Projection splitting algorithms for nonself operators. J. Nonlinear Convex Anal..

[16] Cho S.Y. (2016). Generalized mixed equilibrium and fixed point problems in a Banach space. J. Nonlinear Sci. Appl..

[17] Cho S.Y., Qin X., Yao J.C., Yao Y.H. (2018). Viscosity approximation splitting methods for monotone and nonexpansive operators in Hilbert spaces. J. Nonlinear Convex Anal..

[18] Yao Y.H., Liou Y.C., Yao J.C. (2015). Split common fixed point problem for two quasi-pseudocontractive operators and its algorithm construction. Fixed Point Theory Appl..

[19] Yao Y.H., Liou Y.C., Yao J.C. (2017). Iterative algorithms for the split variational inequality and fixed point problems under nonlinear transformations. J. Nonlinear Sci. Appl..

[20] Yao, Y.H., Yao, J.C., Liou, Y.C., Postolache, M.: Iterative algorithms for split common fixed points of demicontractive operators without priori knowledge of operator norms. Carpathian J. Math. in press

[21] Yao Y.H., Agarwal R.P., Postolache M., Liou Y.C. (2014). Algorithms with strong convergence for the split common solution of the feasibility problem and fixed point problem. Fixed Point Theory Appl..

[22] Byrne C. (2002). Iterative oblique projection onto convex subsets and the split feasibility problem. Inverse Probl..

[23] Censor Y., Segal A. (2009). The split common fixed point problem for directed operators. J. Convex Anal..

[24] Wang F., Xu H. (2011). Cyclic algorithms for split feasibility problems in Hilbert spaces. Nonlinear Anal..

[25] Tang Y.C., Liu L.W. (2016). Several iterative algorithms for solving the split common fixed point problem of directed operators with applications. Optimization.

[26] Moudafi A., Al-Shemas E. (2013). Simultaneous iterative methods for split equality problems and application. Trans. Math. Program. Appl..

[27] Chang S.S., Wang L., Zhao Y. (2017). On a class of split equality fixed point problems in Hilbert spaces. J. Nonlinear Var. Anal..

[28] Tang J., Chang S.S., Dong J. (2017). Split equality fixed point problem for two quasi-asymptotically pseudocontractive mappings. J. Nonlinear Funct. Anal..

[29] Dong Q.L., He S., Zhao J. (2015). Solving the split equality problem without prior knowledge of operator norms. Optimization.

[30] Wu Y., Chen R., Shi L.Y. (2014). Split equality problem and multiple-sets split equality problem for quasi-nonexpansive multi-valued mappings. J. Inequal. Appl..

[31] Byrne C., Moudafi A. (2013). Extensions of the CQ algorithm for the split feasibility and split equality problems. Documents De Travail.

[32] Zhao J. (2015). Solving split equality fixed-point problem of quasi-nonexpansive mappings without prior knowledge of operators norms. Optimization.

[33] Zhao J., He S. (2015). Viscosity approximation methods for split common fixed-point problem of directed operators. Numer. Funct. Anal. Optim..

[34] Zhao J., Wang S. (2016). Viscosity approximation methods for the split equality common fixed point problem of quasi-nonexpansive operators. Acta Math. Sci..

[35] Wang F. (2017). A new iterative method for the split common fixed point problem in Hilbert spaces. Optimization.

[36] Matinez-Yanes C., Xu H.K. (2006). Strong convergence of the CQ method for fixed point processes. Nonlinear Anal..

[37] Hao Y., Cho S.Y., Qin X. (2010). Some weak convergence theorems for a family of asymptotically nonexpansive nonself mappings. Fixed Point Theory Appl..

[38] Bauschke H.H. (1996). The approximation of fixed points of composition of nonexpansive mappings in Hilbert space. J. Math. Anal. Appl..

